# Electrophilic nitroalkene-tocopherol derivatives: synthesis, physicochemical characterization and evaluation of anti-inflammatory signaling responses

**DOI:** 10.1038/s41598-018-31218-7

**Published:** 2018-08-24

**Authors:** Jorge Rodriguez-Duarte, Rosina Dapueto, Germán Galliussi, Lucía Turell, Andrés Kamaid, Nicholas K. H. Khoo, Francisco J. Schopfer, Bruce A. Freeman, Carlos Escande, Carlos Batthyány, Gerardo Ferrer-Sueta, Gloria V. López

**Affiliations:** 1 Laboratory of Vascular Biology and Drug Development, Institut Pasteur de Montevideo, Montevideo, Uruguay; 2 Departmento de Química Orgánica, Facultad de Química, Universidad de la República, Montevideo, Uruguay; 3 INDICYO Program, Institut Pasteur de Montevideo, Montevideo, Uruguay; 4 Laboratorio de Enzimología, Facultad de Ciencias, Universidad de la República, Montevideo, Uruguay; 5 Analytical Biochemistry and Proteomics Unit, Montevideo, Uruguay; 6 Department of Pharmacology and Chemical Biology, University of Pittsburgh, Pittsburgh, PA USA; 7 Laboratory of Metabolic Diseases and Aging, Institut Pasteur de Montevideo, Montevideo, Uruguay; 8 Laboratorio de Fisicoquímica Biológica, Facultad de Ciencias, Universidad de la República, Montevideo, Uruguay

## Abstract

Inflammation plays a major role in the onset and development of chronic non-communicable diseases like obesity, cardiovascular diseases and cancer. Combined, these diseases represent the most common causes of death worldwide, thus development of novel pharmacological approaches is crucial. Electrophilic nitroalkenes derived from fatty acids are formed endogenously and exert anti-inflammatory actions by the modification of proteins involved in inflammation signaling cascades. We have developed novel nitroalkenes derived from α-tocopherol aiming to increase its salutary actions by adding anti-inflammatory properties to a well-known nutraceutical. We synthesized and characterized an α-tocopherol-nitroalkene (NATOH) and two hydrosoluble analogues derived from Trolox (NATxME and NATx0). We analyzed the kinetics of the Michael addition reaction of these compounds with thiols in micellar systems aiming to understand the effect of hydrophobic partition on the reactivity of nitroalkenes. We studied NATxME *in vitro* showing it exerts non-conventional anti-inflammatory responses by inducing Nrf2-Keap1-dependent gene expression and inhibiting the secretion of NF-κB dependent pro-inflammatory cytokines. NATxME was also effective *in vivo*, inhibiting neutrophil recruitment in a zebrafish model of inflammation. This work lays the foundation for the rational design of a new therapeutic strategy for the prevention and treatment of metabolic and inflammation-related diseases.

## Introduction

Chronic non-communicable diseases (CNCD) constitute the major cause of mortality in the world, accounting for 70% of all deaths^1^. Modern life styles, which include sedentarism and high-caloric diets concomitant with a high daily sugar intake has led to a dramatic increase in the incidence of these pathologies^2^. Among cardiovascular diseases, atherosclerosis is a major health issue, leading to 30 to 50% of deaths worldwide^2^. In the last decade, low grade, chronic and sterile inflammation have been associated with the activation of different cellular signaling pathways (mainly NF-κB and NLRP3 inflammasome activation) and has appeared as an important player in the pathogenesis of these chronic diseases^3–9^. The inflammatory response promotes the olefinic nitration of unsaturated fatty acids such as conjugated linoleic acid, resulting in the formation of nitroalkenes^10,11^. These molecules exert pleiotropic anti-inflammatory responses due to the post-translational modification of functionally-significant proteins^12^ that are critically involved in regulating inflammatory and metabolic signaling. Nitroalkenes are activators of Peroxisome-Proliferator-Activated-Receptor-gamma (PPAR-γ), Heat-Shock-Response (HSR) and nuclear factor erythroid 2-related factor 2/Kelch ECH associating protein 1 (Nrf2-Keap1), whilst they inhibit pro-inflammatory responses regulated by the transcription factor NF-κB^10,13–17^. Due to these interesting signaling properties, unsaturated fatty acid nitroalkene derivatives have been tested in preclinical animal models of CNCD. Their principal pharmacological applications include the treatment of atherosclerosis, systemic and pulmonary hypertension and diabetes. In fact, nitrated unsaturated fatty acids have shown promising results in animal models of all these diseases^18–23^. However, nitroalkenes derived from fatty acids have metabolic disadvantages due to their metabolism and inactivation via β-oxidation, olefin saturation and thiol conjugation^24^. These limitations, as well as other factors, affirm the need for novel approaches to improve efficacy while maintaining similar anti-inflammatory activity.

Aiming to develop novel pharmacological compounds for the prevention and treatment of inflammatory and metabolic diseases (e.g. cardiovascular, hypertension, obesity-induced insulin resistance, type II diabetes), we have developed nitroalkene analogues of α-tocopherol as novel dual anti-oxidant and anti-inflammatory mediators.

**Figure 1:**
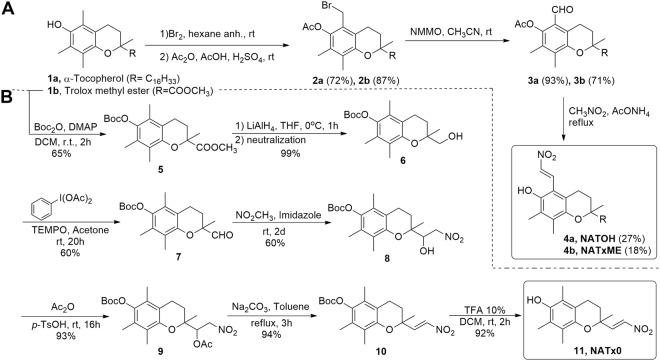
Synthesis of novel nitroalkene analogues of α-tocopherol. (**A**) Synthesis of NATOH and NATxME; (**B**) Synthesis of NATx0.

Dietary α-tocopherol, the most biologically active form of vitamin E, is preferentially incorporated into nascent lipoproteins by the action of α-tocopherol transfer protein (α -TTP) in the liver, and from there distributed to the organism. Thus, we took advantage of this scaffold and used the lipoproteins to transport the nitroalkene-tocopherol analogue as cargo. The nitroalkene derivative of α-tocopherol (NATOH, Fig. 1) was designed to be distributed by lipoproteins, reaching the vasculature and, importantly, the atheroma plaque, the characteristic lesion of atherosclerosis.

In parallel, we designed and synthesized nitroalkenes derived from Trolox™ (6-hydroxy-2,5,7,8-tetramethylchroman-2-carboxylic acid), a water-soluble analogue of α-tocopherol (NATxME and NATx0, Fig. 1), aiming to develop other non-conventional anti-inflammatory compounds with different physicochemical and absorption, distribution, metabolization and excretion properties. NATxME possesses the nitroalkenyl group in the same position as NATOH, meanwhile NATx0 have this electrophilic group in another position, not conjugated with the aromatic ring. The structural differences allow us to study how the position of the nitroalkene group may modulate their electrophilicity and therefore their biological effects.

The predominant mechanism by which nitroalkenes exert signaling actions is a consequence of the Michael addition of nucleophiles such as cysteine and, at higher concentrations, histidine residues in proteins^12,25^. Such reaction involves a charged transition state that is expected to be disfavored in hydrophobic media^26^. Therefore, reaction kinetics in aqueous solution only partially describes the fate and target selectivity of nitroalkenes *in vivo*. In that respect, the study of the reactivity of NATOH and its less hydrophobic analogues represents an opportunity to assess the effect of membrane partition on the reactivity and selectivity of electrophiles.

Additionally, we evaluated the anti-inflammatory properties for nitroalkenyl-containing compounds in cell culture, and to move further, we examined whether these hybrid compounds may exert their protective effects *in vivo* using a zebrafish model of inflammation.

In summary, we performed a physicochemical study to understand determinants of nitroalkenes reactivity, and tested their anti-inflammatory actions *in vitro* and *in vivo*. This study established foundations to rationally modify a specific synthetic molecule in order to improve its pharmacological properties.

## Results

### Synthesis of nitroalkene analogues of α-tocopherol and Trolox: NATOH, NATxME, NATx0

In the first two compounds (Fig. 1A), the nitroalkenyl group is located on the 5-position of the chroman ring of α-tocopherol **1a** or on its water soluble analogue, methyl 6-hydroxy-2,5,7,8-tetramethylchromancarboxylate (Trolox methyl ester) **1b**, obtained as described^27^. The synthetic route by which these derivatives were obtained is depicted in Fig. 1. Alpha-tocopherol or Trolox methyl ester were transformed to the corresponding bromoacetate by treatment with bromine followed by *O*-acetylation^28,29^. Then, the 6-*O*-acetyl-5a-bromo derivative **2** oxidation with *N*-methylmorpholine *N*-oxide yields the corresponding aldehyde **3** as described in the literature^30,31^. Finally, condensation of **3** with nitromethane, in the presence of ammonium acetate, gave the desired nitroalkene **4a** (NATOH) or **4b** (NATxME) (Fig. 1A)^32^. Due to the coupling constant of the doublets of the alkene observed in the ^1^H NMR spectra, the configuration of both nitroalkenes is consistent with an *E* isomer (Fig. 1A).

Additionally, a derivative with the nitroalkenyl group located on the side chain at the 2-position of the chroman ring was synthesized as described in Fig. 1B. The phenolic group of **1b** was protected by treatment with di-*tert*-butyl dicarbonate to produce the ester **5**^33^. Then, the methyl ester was reduced to a primary alcohol^34^ and subsequently oxidized to produce the aldehyde derivative** 7**. The β-nitro alcohol intermediate **8** was obtained via condensation of **7** with nitromethane in the presence of imidazole, followed by acetylation and elimination to render the nitroalkene **10** with almost quantitative yields. Finally, phenol deprotection gave the desired product **11** (NATx0, Fig. 1B) with an *E* configuration as suggested by the coupling constant observed in the ^1^H NMR spectra.

### Electrophilic behavior of NATOH, NATxME and NATx0

**Figure 2:**
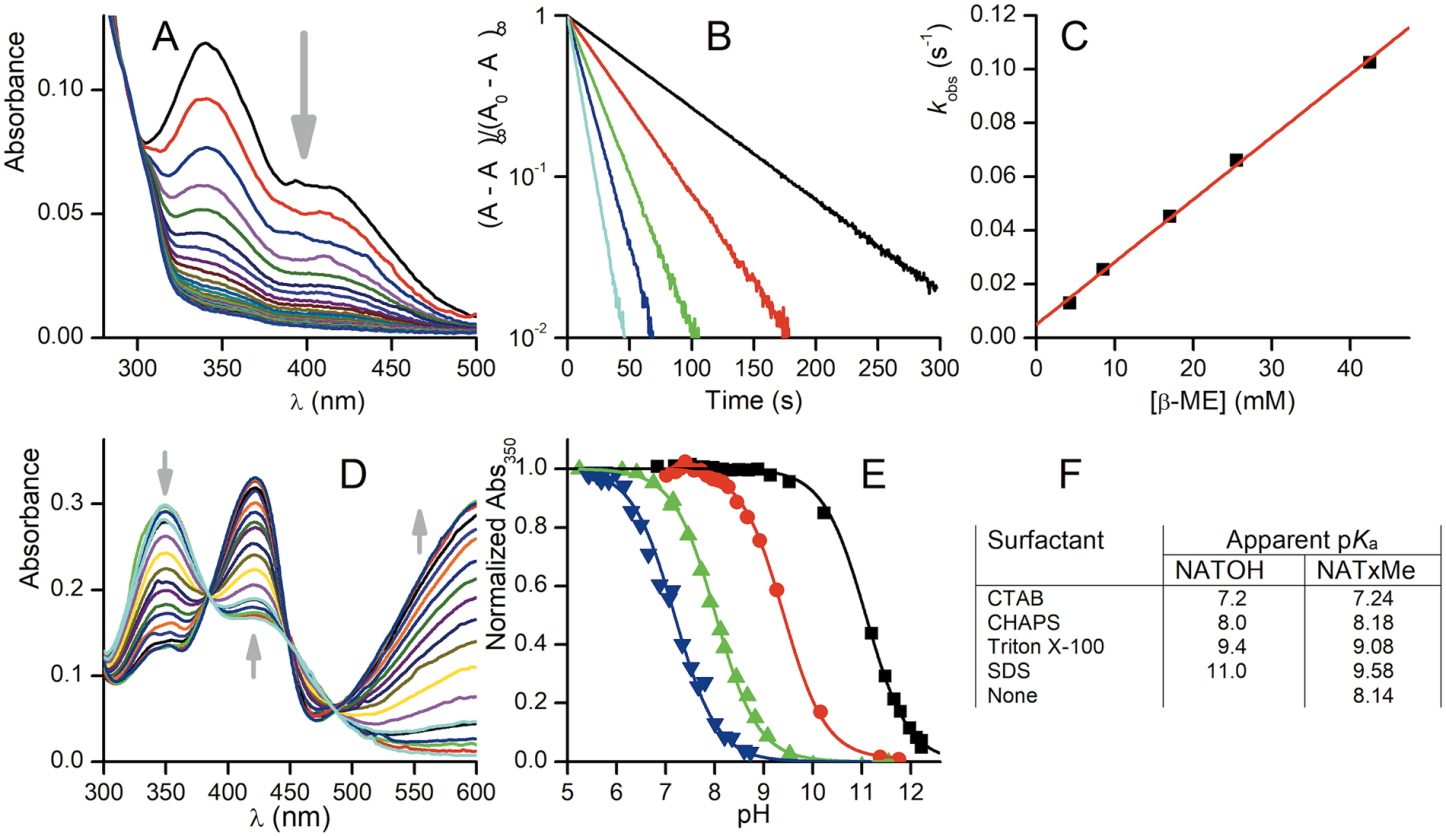
Reaction of synthetic nitroalkenes with nucleophiles in different environments. (**A**) Spectral change upon reaction of 10 µM NATOH with 0.1 mM βME in TMA20 buffer at pH 8.1 containing 2.5 g/L SDS. **(B)** First-order plots obtained at different concentrations of βME measuring absorbance at 350 nm. **(C)** Second-order plot for the same experiment, linear fit parameters k_f_ (slope) = 2.3 M^−1^ s^−1^, k_r_ (intercept) = 5 × 10^−3^ s^−1^. **(D)** Spectral change upon titration of NATOH in CHAPS micelles. The arrows indicate the change upon increase in pH of the bulk solution. **(E)** Titration curves of NATOH at 350 nm in TMA20 buffer and different micelle suspensions, from left to right, CTAB, CHAPS, Triton X-100 and SDS. The absorbance was normalized to the acidic and alkaline extrapolated values of the fit. **(F)** Apparent p*K*_*a*_ results of NATOH and NATxME in different micellar media.

The accepted mode of action driving the biological activity of nitroalkenes is by addition reactions with nucleophiles, mainly thiols^12,26^. We approached the kinetic characterization of the addition of thiols to our synthetic nitroalkenes taking advantage of their optical and partition properties. The three novel nitroalkenes synthesized were observed to react with glutathione (GSH) and β-mercaptoethanol (βME). In the case of NATOH and NATxME the addition reaction disrupts the chromophore formed by the nitroalkene conjugated to the aromatic chroman, leading to a dose dependent decrease in UV-Visible absorption (Fig. 2A and Figure S1). This decrease in absorbance was used to monitor the reaction at the λ_max_ 350 nm. In the case of NATx0 there is no disruption of the chromophore because the nitroalkene is not conjugated to the chromane. Eventhough the reaction was followed spectrophotometrically at 285 nm (consumption of the nitroalkene) and at 260 nm (production of the adduct) (Figure S1B)^16^. This reaction shows similar spectrophotometric behaviors as the one exerted by nitrated unsaturated fatty acids with nucleophiles^16^.

The reaction is first order in nitroalkene (Fig. 2B) and first order in thiol with a nonzero intercept (Fig. 2C), implying a reversible Michael addition reaction, as expected^26^. The expected trend of the apparent second-order rate constants in neutral aqueous solution is higher for GSH than for βME, given that GSH is more acidic than βME and therefore a higher fraction of the more reactive thiolate is available. That trend is observed in the absence of surfactants with NATxME and NATx0 (Table 1) and also with cationic CTAB micelles with NATOH. Nevertheless, the trend is mostly absent in zwitterionic CHAPS micelles and reversed in neutral (Triton X-100) and anionic (SDS) micelles (Table 1).

**Table 1:** Rate constants of nucleophilic addition of NATOH and NATxME to GSH and β-ME in micellar suspension and aqueous solution.

Surfactant	NATOH			
	Electrophilic reactivity			
	Thiol	pH	*k*_f_ (M^−1^ s^−1^)	*k*_r_ (s^−1^)
CTAB	GSH	7.1	576	0.36
	β-ME	7	343	0.13
CHAPS	GSH	6.7	34	1.7 × 10^−2^
		7.0	100	4 × 10^−2^
		7.3	148	6.2 × 10^−2^
	β-ME	6.9	53	9.9 × 10^−3^
		7.1	83	1.5 × 10^−2^
		8.3	383	0.15
Triton X-100	GSH	7.1	0.8	N.D.
	β-ME	7	6.5	1.8 × 10^−3^
SDS	GSH	7.2	0.2	
	β-ME	7.5	0.63	3 × 10^−4^
		7.8	1.0	4.9 × 10^−4^
		8.1	2.3	5 × 10^−3^
	**NATxMe**			
None	GSH	7.1	1370	N.D.
	β-ME	7.1	349	
	**NATx0**			
None	GSH	7.1	71	N.D.
	β-ME	7.1	21	
	GAPDH	7.4	220	

Addition rate constant of NATx0 to GSH, β-ME and GAPDH.

In both NATOH and NATxME, the chromophore contains an ionizable phenol, thus the acid-base equilibrium of the phenol also conveys a large change in absorbance (Fig. 2D). The titration of both nitroalkenes with NaOH allows the determination of their apparent p*K*_a_ in the same micellar systems and thus, reflects the bulk pH as perceived in the micelle interior. The p*K*_a_ values for NATOH span a range of 3.8 units with the expected trend of most acidic in cationic CTAB micelles and least acidic in anionic SDS (Fig. 2E). The trend is similar for NATxME but within a narrower range of nearly 2.4 units (Fig. 2E,F). The p*K*_a_ of NATxME was determined both in micellar suspension and in aqueous solution in an attempt to separate the effect of apparent pH from the effects of partitioning of the nucleophiles and on the reaction rate itself. We observed (Fig. 2F and Figure S2) that the p*K*_a_ of NATxME in aqueous solution and in zwitterionic CHAPS micelles are essentially equal and very similar to the p*K*_a_ of NATOH in CHAPS micelles (8.14, 8.18 and 8.0, respectively). Then, and as a first approximation, CHAPS micelles cancel out the effect of surface pH as perceived by the nitroalkene.

Comparing the reaction of βME with NATOH in CHAPS and with NATxME in aqueous solution, we found a four-fold decrease in the rate constant at pH 7.1. If this comparison is made for the corresponding reactions with GSH, the rate constant is more than ten-fold lower in the CHAPS micelles. The difference may be explained in terms of the extra charges in GSH that further destabilize its presence in the hydrophobic core of the micelles. Furthermore, neutral and anionic micelles provoke a further decrease of two to three orders of magnitude in the rate constant for thiol reaction with NATOH and NATxME, and in all cases, the decrease is most marked for GSH (Table 1).

### Anti-inflammatory properties of NATxME *in vitro* and *invivo*

Since NATxME was shown to be the most electrophilic compound in all our experimental setups (see Table 1) and it is also water soluble in all concentrations used (important property for the *in vivo* model used herein, see below), we decided to initially study the biological properties of this compound, leaving the others for future works.

### NATxME effects in macrophages

Since our main goal is to target the prevention and treatment of inflammation related diseases, we focused first on two of the main inflammatory signaling pathways known to be modulated by nitro-fatty acids: NF-кB-dependent expression of pro-inflammatory cytokines and Nrf2-Keap1 regulated gene expression^35^. We analyzed NATxME effects in macrophages (RAW 264.7 cells), since they are key players in inflammatory responses and disease pathogenesis^6^, and chose NATxME as a starting molecule for characterization, given its electrophilic and hydrophilic properties.

**Figure 3:**
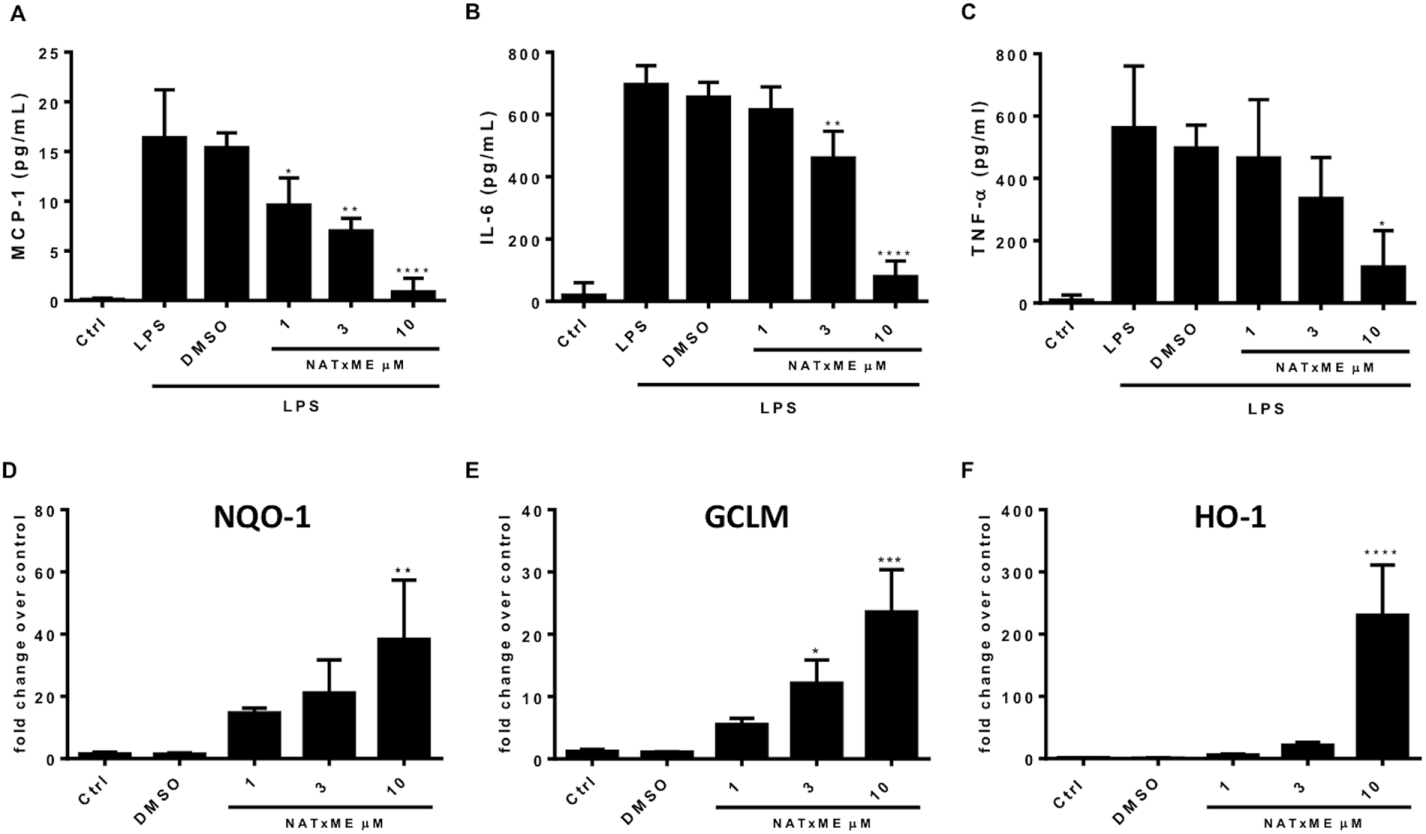
NATxME anti-inflammatory and cytoprotective effects. NATxME inhibits the secretion of pro-inflammatory cytokines (**A**–**C**). RAW 264.7 macrophages were treated with NATxME 1, 3 and 10 µM for 2 h and then stimulated with LPS (50 ng/ml) for 18 h. The secretion of inflammatory cytokines MCP-1 **(A)**, IL-6 **(B)** and TNF-α **(C)** was analyzed by ELISA in the supernatant. Values are shown as mean ± SD. Statistical analysis: one-way ANOVA Bonferroni’s multiple comparison test. (A) * = 0.0294; ** = 0.0017; **** < 0.0001. (B) ** = 0.013; **** < 0.0001. (C) * = 0.0131. *vs.* LPS. NATxME induces the Nrf2-Keap-1 system (**D**–**F**). Raw 264.7 macrophages were treated with NATxME 1, 3 and 10 µM for 5 h. The expression of NQO1 **(D)**, GCLM **(E)** and HO-1 **(F)** were analyzed by quantitative RT-PCR. Values are shown as mean ± SD. Statistical analysis: one-way ANOVA Bonferroni’s multiple comparison test. (D) ** = 0.0038. (E) * = 0.0310; *** = 0.0001. (F) *** = 0.0001. vs control.

We started evaluating the doses at which NATxME should be used in culture, measuring the cytotoxicity of NATxME in RAW 264.7 macrophages, and found that the IC_50_ was 25 µM (Figure S3). Then, to determine if NATxME modulates the NF-кB signaling cascade, we incubated RAW 264.7 macrophages with NATxME (1, 3 and 10 µM; Fig. 3) for 2 h before cells were stimulated with lipopolysaccharides (LPS) and we compared these results to the vehicle control (DMSO). Cell supernatant was then analyzed by ELISA (Enzyme-Linked ImmunoSorbent Assay) for NF-κB-dependent cytokine secretion (Fig. 3A–C). We observed a marked inhibition of monocyte chemoattractant protein-1 (MCP-1), Interleukin-6 (IL-6) and tumor necrosis factor alpha (TNF-α)  secretion. These effects were dose-dependent, reaching ∼80% inhibition at 10 µM NATxME.

Similarly, we used the same NATxME doses for analyzing Nrf2-Keap1 regulated gene expression. In this case, the expression of phase 2 detoxification genes were measured by qRT-PCR. Remarkably, all three transcripts: NAD(P)H dehydrogenase quinone 1 (NQO-1), glutamate-cysteine ligase modifier subunit (GCLM) and heme oxygenase-1 (HO-1) were significantly up-regulated by NATxME in a dose-dependent manner (Fig. 3D–F).

### NATxME inhibits neutrophil recruitment in zebrafish *in vivo*

**Figure 4:**
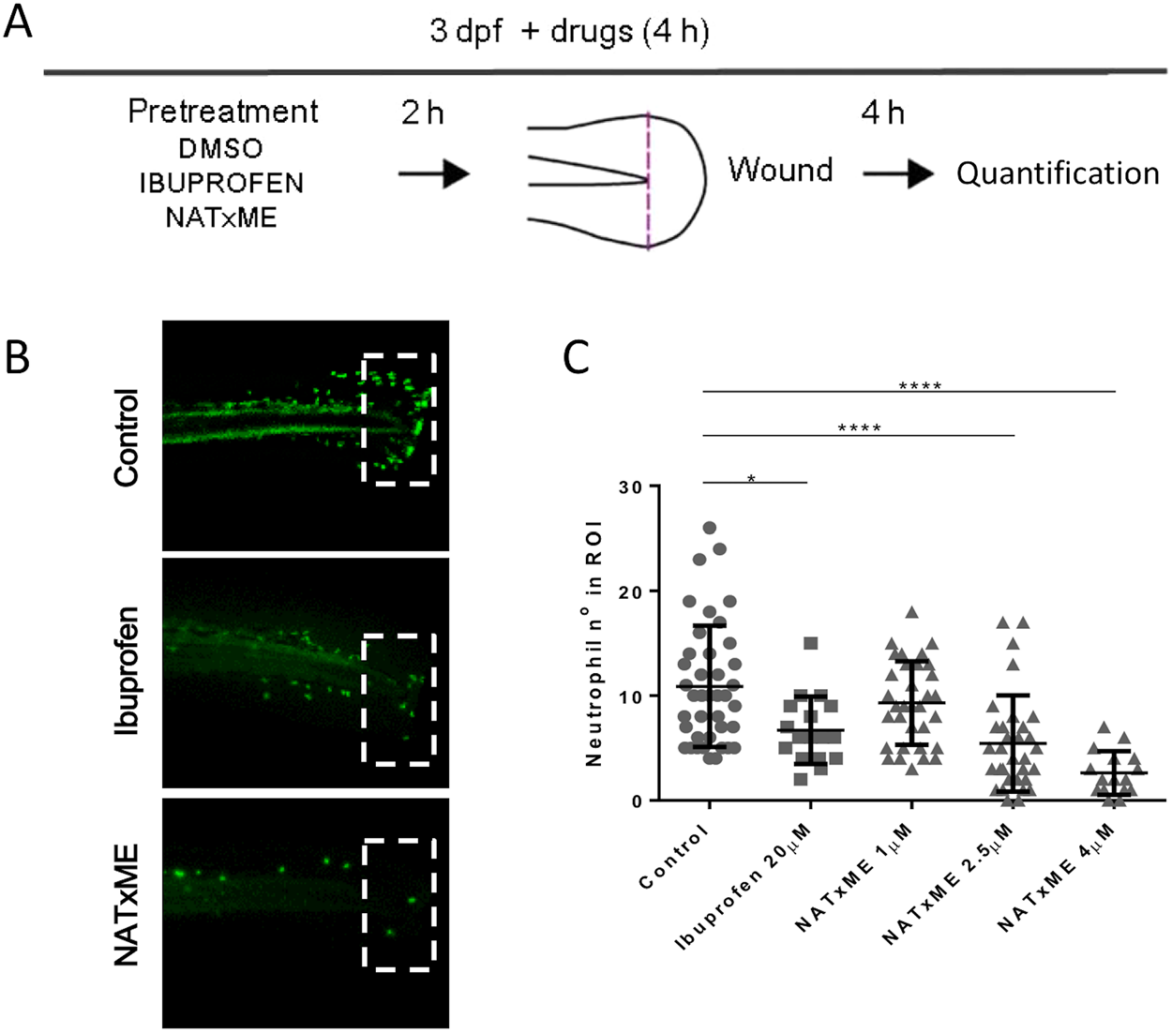
NATxME inhibits neutrophil recruitment in zebrafish. (**A**) Diagram of acute inflammation assay. At 3 dpf zebrafish larvae were pre-treated for 2 h with DMSO, ibuprofen or NATxME. Wounding was performed in tail fins by transection with a scapel, and larvae were incubated again in the presence of drugs. Neutrophils at the site of injury were imaged and counted 4 h post transection. **(B)** Representative pictures used for quantification, corresponding to DMSO, 20 µM ibuprofen and 4 µM NATxME. Dotted rectangles indicate the wounded region of interest (ROI) where neutrophils were counted. **(C)** Quantification of neutrophil recruitment to wounded fins at 4 h under different treatments. Larvae number were: DMSO^40^, 20 µM ibuprofen^17^, 1 µM NATxME^33^, 2.5 µM NATxME^32^, 4 µM NATxME^17^. Statistical analysis: one-way ANOVA Bonferroni’s multiple comparison test, * = 0.0164; **** < 0.0001.

We then tested the anti-inflammatory effects of NATxME *in vivo*, using transgenic zebrafish larvae (Tg:mpx:GFP) in which neutrophils express Green Fluorescent Protein (GFP). This is a widely validated experimental setup in which the inflammatory response can be followed *in vivo* by directly measuring neutrophil recruitment in response to a localized mechanical injury^36^. Figure 4A shows a diagram of these experiments, in which Tg:mpx:GFP larvae at 3 days post-fertilization (3 dpf) were incubated for 2 h with DMSO (vehicle), ibuprofen (positive control)^37^ or NATxME. Then, their caudal fins were wounded by transection with a scalpel and incubated again for 4 h. At this time, we counted the number of neutrophils recruited to the site of injury, as it is when they reach the maximum^36^. As expected, control (DMSO) larvae displayed the typical neutrophil recruitment (mean = 10.9, n = 40) and the anti-inflammatory ibuprofen (20 µM) significantly reduced this number (mean = 6; n = 17; p = 0.0164). Remarkably, NATxME also significantly reduced the number of neutrophils recruited, even at lower concentrations (2.5 µM; mean = 5.4; n = 32, p = 0.0001; Fig. 4C). This effect was dose dependent and at 4 µM, NATxME showed a more pronounced decrease in neutrophil number (mean = 2; n = 16, p = 0.0001; Fig. 4C).

## Discussion

The aim of the present work was to develop novel nitroalkene analogues of α-tocopherol designed for the prevention and treatment of inflammation related diseases. These hybrid compounds were synthesized by incorporating a nitroalkenyl moiety, with recognized anti-inflammatory properties, to well-known nutraceuticals: α-tocopherol or trolox, (Fig. 1). By using this strategy, we synthesized two kinds of nitroalkene derivatives, one hydrophobic, designed to be incorporated into lipoproteins (NATOH, Fig. 1A) and two water soluble (NATxME, Fig. 1A; and NATx0, Fig. 1B). In fact, due to structural differences, these compounds exhibited different electrophiliic behavior as discussed below.

Our kinetic studies showed that the addition reaction between nitroalkenes and thiols, differ significantly when the reaction is carried out in a microheterogeneous system such as micellar dispersion (Fig. 2 and Table 1)^38^. The expected trend of the reactivity in aqueous solution is that GSH should be faster than βME at neutral pH, given that the thiol of GSH is a stronger acid than that of βME and therefore a higher fraction of thiolate is available (Table 1).

The effects of the micelles on the electrophilic reactions in general are complex and multifactorial, they have been studied through numerous experimental approaches for a long time^39^. In this work, we attempted to dissect the different effects taking advantage of the acid-base and optical properties of NATOH and NATxME, which share the same functional group and chromophore but differ in their water solubility. The micelle dispersion exerts at least three effects on the reaction rate. First, the pH at the surfactant-water interface is affected by the charge of the head groups. For instance, anionic SDS micelles attract H^+^ ions causing a drop in the interfacial pH relative to the bulk solution. Second, the transition state of the addition reaction has a negative charge that is also differentially stabilized by the charge of the micelle and, in all cases, destabilized by the lower dielectric constant of the hydrophobic interior. Therefore, the reaction is expected to be slower due to the hydrophobic medium and more so if the charge of the micelle is negative. Finally, the charge of the nucleophile affects its partition into the hydrophobic interior of the micelle. In this aspect, both GSH and βME as thiolates are anionic nucleophiles, but GSH has the additional charges of the amino and the two carboxylate groups, making it much less stable in hydrophobic environments. The first two effects are shared by both thiols as the reactions are slowed down by the hydrophobic environment of the micelle and similarly affected by the surface pH of charged micelles (Table 1).

The differential partitioning of the thiolates favors βME more than GSH. The narrow range of p*K*_a_ observed during the titration of NATxME in comparison with NATOH, may be due to the partitioning of the two nitroalkenes, which differ in their hydrophobicity (Fig. 2F). Thus, NATOH, which has a trimethyldecyl side chain of tocopherol, is expected to partition almost quantitatively into the hydrophobic phase. On the other hand, NATxME is partially water-soluble thus the measured p*K*_a_ most likely reflects the mixture of NATxME molecules both inside and outside of micelles. The rate constant at pH 7.1 of the reactions of βME with NATxME in an aqueous solution was higher than that of NATOH in CHAPS micelles (349 *vs.* 83 M^−1^ s^−1^, Table 1). This is probably due to the destabilization of both the thiolate nucleophile and the anionic transition state of the reaction. The same comparison for the reactions of GSH yields a more dramatic drop in rate constants (1370 *vs.* 100 M^−1^ s^−1^, Table 1) underscoring the relative importance of the charge of the nucleophile.

In the context of the biological activity of NATOH, its partition to hydrophobic compartments (such as membranes, lipoproteins or the fat storage area of adipocytes) will dictate a significant portion of its electrophilic reactivity. The surface of such compartments is composed of anionic, neutral and zwitterionic lipids that will significantly diminish the rate of reaction with charged thiols such as GSH, thus preventing its rapid excretion. On the other hand, a protein thiol able to interact with a lipid–water interface and, if both the attacking thiolate and the transition state of the addition reaction, are stabilized by the protein environment, the result would be a specific interaction able to yield biological effects even at low concentrations of the electrophile.

NATx0 showed relatively slower reactions with both β-ME and GSH (21 and 71 M^−1^ s^−1^, respectively) compared to NATxME. The β-carbons of NATxME and NATOH resulted more electrophilic than those of NATx0 (Table 1). NATx0 is similar to nitro fatty acids, and the second order rate constants with GSH are consistently similar to those of nitro oleic acid (64 M^−1^ s^−1^, pH 7.4, 25 °C) and nitro conjugated linoleic acid (34 M^−1^ s^−1^, pH 7.4, 25 °C)^26^. Even though NATx0 is the slowest electrophile studied herein, its reaction with GAPDH is significantly faster than with GSH (Table 1, Figure S4) pointing to the importance of a protein active site containing an acidic thiol and cationic residues in the vicinity that could stabilize the anionic transition state.

To sum up the reactivity results, the effects of the hydrophobic partition and the charge at the lipid-water interface may protect electrophiles such as NATOH from addition to GSH and other unspecific and water-soluble thiols. Conversely, a cysteine residue in a protein able to interact with a lipidic microenvironment, and with cationic residues that could stabilize the transition state of the addition, may represent a selectivity mechanism of an otherwise general electrophile.

Fatty acid nitroalkenes are pleiotropic modulators of anti-inflammatory and anti-oxidant cell signaling responses *in vitro* and *in vivo*^28^. We showed that the new compound NATxME displayed two of these important functions in a macrophage cell line, the RAW264.7, which is relevant because macrophages act as major players in inflammatory responses^6^. First, when the RAW264.7 cells were activated with LPS, NATxME inhibited the secretion of pro-inflammatory citokines MCP-1, IL-6 and tumor TNF-α. This is a well characterized macrophage response mediated by NF-κB, critical for initiating inflammation responses^17^. Second, we showed that NATxME induced the transcription of three phase II enzymes regulated by the antioxidant-response element, regulated by Nrf2^29^. Because oxidative stress and inflammation are two intertwined processes, Nrf2-regulated genes are critical in mediating cell defense against oxidative stress by expressing antioxidant and detoxifying enzymes that limit inflammation and reactive species (RS)-induced damage^40^.

In addition to this, we went a step further and analyzed NATxME effects on a vertebrate model of acute sterile inflammation *in vivo*. Using transgenic zebrafish larvae that express GFP in neutrophils^36^, we were able to directly follow neutrophil recruitment to a specific injured site, *in vivo* and in real time. This model has been extensively characterized in the last ten years, demonstrating conservation of cellular and molecular mechanisms with mammalian inflammatory processes^35,36,41^ and allowing discovery and testing of anti-inflammatory compounds^42,43^.

Remarkably, in this work we showed that NATxME greatly inhibited the recruitment of neutrophils to the injured tissue, further demonstrating its anti-inflammatory properties. The precise cellular and molecular mechanisms involved in this system *in vivo* remains to be determined and will be the focus of future work. Nonetheless, it is interesting to note that several studies in zebrafish have shown the requirement of NF-κB activation and interleukin secretion at the site of injury as a critical step regulating leucocyte recruitment^44–46^. Also, another nitro-fatty acid, nitrooleic acid (OA-NO_2_), has been shown to activate Nrf2 in a transgenic zebrafish that expresses green fluorescent protein (GFP) in response to Nrf2 activators^47^.

Thus, our findings highlight these tocopherol-nitroalkene analogues as lead compounds for the development of novel drugs for the prevention and treatment of inflammation related diseases. Moreover, our work set the foundations to modify a specific nitroalkene-based synthetic compound in order to improve its pharmacological properties to better tackle each specific inflammatory related disease.

## Experimental Section

### Chemistry

Argon and nitrogen were purchased from Linde Uruguay (Montevideo, Uruguay). Other chemicals were purchased from Sigma (St. Louis, MO), Aldrich (Milwaukee, WI) or Applichem (Germany) at the highest purity available. The tocopherol used along the study was always (±)-α-TOH (Sigma Aldrich, St. Louis, USA). Compound **1b** was synthesized according to literature methods^27^. ^1^H NMR and ^13^C NMR spectra were recorded on a Bruker DPX-400 instrument, with CDCl_3_ as solvent and tetramethylsilane as the internal reference. Electron impact (EI) and electrospray (ES+) mass spectra were obtained at 70 eV on a Shimadzu GC-MS QP 1100 EX or on a Hewlett Packard 1100 MSD spectrometer, respectively. TLC was carried out on Alugram® Sil G/UV254 or Aluminum oxide on polyester plates. Column chromatography (CC) was carried out on silica gel (Merck, 60–230 mesh) or aluminum oxide (Merck, 70–230 mesh). All solvents were of anhydrous quality purchased from Aldrich Chemical Co. and used as received.

### 5-(bromomethyl)-2,7,8-trimethyl-2-(4,8,12-trimethyltridecyl)-3,4-dihydro-2H-chromen-6-yl acetate (**2a**)

To a solution of (±)-α-tocopherol (**1a**, 1.38 g, 3.20 mmol) in dry n-hexane (25.0 mL) a solution of bromine (1.05 equiv) in n-hexane (10.0 mL) was added dropwise at room temperature. The mixture was stirred for 3 h. Solvent and remaining bromine were removed *in vacuo* at room temperature. In the same flask was then carried out the acetylation reaction to bromomethyl derivative obtained as described above, were added CH_2_Cl_2_ (12.0 mL), AcOH (12.0 mL), Ac_2_O (2.2 mL) and H_2_SO_4_ (0.2 mL). The dark mixture was stirred overnight at room temperature. Then water was added and CH_2_Cl_2_ evaporated. The aqueous phase was extracted with hexane (3 × 100 mL). The combined organic extracts were subsequently washed to neutrality with water, dried over Na_2_SO_4_ and concentrated to dryness. Purification by column chromatography (Hex:EtOAc, 10:1) afforded **2a** (1.24 g, 72% yield from 1a) as yellow dense oil. Analytical data are consistent with those given in the literature^31,40,48^. ^1^H NMR (400 MHz, CDCl_3_): δ 4.42 (bs, 2 H), 2.80 (t, *J* 6.6 Hz, 2 H), 2.41 (s, 3 H), 2.14 (s, 3 H), 2.04 (s, 3 H), 1.91–1.77 (m, 2 H), 1.59–1.07 (m, 24 H), 0.90–0.86 (m, 12 H).

### Methyl 6-acetoxy-5-bromomethyl-2,7,8-trimethylchroman-2-carboxylate (**2b**)

A round-bottom flask was charged with 1.58 g (6.00 mmol) of **1b**, 33.0 mL of CH_2_Cl_2_. The mixture was stirred at room temperature in the dark, and a solution of 0.3 mL (6.00 mmol) of bromine in 4.5 mL of CH_2_Cl_2_ was added dropwise. Stirring continued at room temperature for 2 h after completion of the bromine addition; the resulting solution was dark, but no bromine color or vapor was detectable. The mixture was purged with a stream of nitrogen to remove most of the HBr present, then stripped to dryness under reduced pressure. The crude intermediate product was dissolved with 13 mL of CH_2_Cl_2_, and treated with 11.0 mL of glacial acetic acid, 3.0 mL of acetic anhydride, and 1 drop of concentrated sulfuric acid. After stirring overnight at room temperature, the mixture was treated with 60.0 mL of water and stirred for 1 h. The mixture was transferred to a separatory funnel and the layers were separated. The aqueous layer was extracted with CH_2_Cl_2_. The combined organic layers were washed with a saturated solution of NaCl (brine), dried (Na_2_SO_4_), and solvent evaporated under reduced pressure. The crude residue purified by column chromatography (Hex:Et_2_O, 7:3), affording **2b** (2.0 g, 87% yield) as white solid. Analytical data are consistent with those given in the literature^30^. ^1^H NMR (400 MHz, CDCl_3_): δ 4.48–4.18 (bd, 2 H), 3.69 (s, 3 H); 2.88–2.81 (m, 1 H), 2.68–2.59 (m, 1 H), 2.49–2.42(m, 1 H), 2.38 (s, 3 H), 2.19 (s, 3 H), 2.02 (s, 3 H), 1.93–1.86 (m, 1 H), 1.62 (s, 3 H).

### 5-formyl-2,7,8-trimethyl-2-(4,8,12-trimethyltridecyl)-3,4-dihydro-2H-chromen-6-yl acetate (**3a**)

To a solution of **2a** (1.24 g, 2.25 mmol) in dry acetonitrile (20 mL), NMMO (*N*-methylmorpholine-*N*-oxide, 1.05 g, 9.00 mmol) was added. After stirring overnight at room temperature, the solvent was evaporated under reduced pressure and the crude residue purified by column chromatography (Hex:EtOAc, 15:1), affording **3a** (1.01 g, 93%) as yellow dense oil. Analytical data are consistent with those given in the literature^31,40,48^. ^1^H NMR (400 MHz, CDCl_3_): δ 10.29 (s, 1 H), 3.12 (m, 2 H), 2.39 (s, 3 H), 2.21 (s, 3 H), 2.09 (s, 3 H, ArCH3), 2.09–1.63 (m, 2 H), 1.41–1.08 (m, 24 H), 0.90–0.86 (m, 12 H).

### Methyl 6-acetoxy-5-formyl-2,7,8-trimethylchroman carboxylate (**3b**)

A solution of 2.00 g (5.20 mmol) of bromoacetate **2b** in 17 mL of dry acetonitrile treated with 1.58 g (15.60 mmol, 3 equiv) of NMMO was stirred at room temperature for 18 h. The mixture was concentrated to about 5 mL under reduced pressure. This concentrated solution was poured into water and extracted with ethyl acetate. The combined organic layers were washed with 5% HCl and with brine, dried (Na_2_SO_4_), and solvent evaporated under reduced pressure. The crude residue purified by column chromatography (Hex:Et_2_O, 9:1), affording **3b** (1.2 g, 71% yield) as orange oil that crystallized on stand. Analytical data are consistent with those given in the literature^30^. ^1^H NMR (400 MHz, CDCl_3_): δ 10.22 (s, 1 H), 3.71 (s, 3 H), 3.29–3.22 (m, 1 H), 2.92–2.83 (m, 1 H), 2.46–2.40 (m, 1 H), 2.38 (s, 3 H), 2.28 (s, 3 H), 2.09 (s, 3 H), 1.88–1.80 (m, 1 H), 1.64 (s, 3 H).

### 2,7,8-trimethyl-5-((E)-2-nitrovinyl)-2-(4,8,12-trimethyltridecyl)-3,4-dihydro-2H-chromen-6-ol (**4a**, **NATOH**)

Aldehyde **3a** (0.15 g, 0.30 mmol) was added in a mixture of 1.2 mL anhydrous CH_3_NO_2_ and equivalent amount of NH_4_CH_3_COO. The mixture was stirred at 100 °C for 2 h. The solvent is then evaporated under reduced pressure and H_2_O and Et_2_O were added. The organic layer was washed with H_2_O (2 × 50 mL), 3 N HCl (2 × ·25 mL), and saturated aqueous NaCl, dried, and the solvent was evaporated. The crude residue purified by column chromatography (Hex:EtOAc, 9:1), affording **NATOH** (0.04 g, 27%) as yellow dense oil. ^1^H NMR (400 MHz, CDCl_3_): δ 8.31 (d, *J* 13.4 Hz, 2 H), 8.14 (d, *J* 13.4 Hz, 2 H), 2.88 (t, *J* 2.9 Hz, 2 H), 2.21 (s, 6 H), 1.92–1.80 (m, 2 H), 1.63–1.07 (m, 24 H), 0.90–0.85 (m, 12 H). ^13^C NMR (125 MHz CDCl_3_): δ 148.1, 146.5, 139.8, 132.7, 131.3, 120.6, 119.4, 112.8, 74.9, 39.3 (2 C), 36.8 (4 C), 31.7 (2 C), 30.6, 28.1, 23.8 (2 C), 22.3 (2 C), 20.7 (2 C), 20.4, 19.2 (2 C), 11.5 (2 C). HRMS m/z calcd for C_30_H_49_NO_4_ [M − H]^+^ 486.3589, found 486.3587.

### Methyl (E)-6-hydroxy-2,7,8-trimethyl-5-(2-nitrovinyl)chroman-2-carboxylate (**4b**, **NATxME**)

Aldehyde **3b** (0.18 g, 0.56 mmol) was added in a mixture of 2.5 mL anhydrous CH_3_NO_2_ and an equivalent amount of NH_4_CH_3_COO. The mixture was stirred at 100 °C for 2 h. The solvent was then evaporated under reduced pressure and H_2_O and Et_2_O were added. The organic layer was washed with H_2_O (2 × 50 mL), 3 N HCl (2 × 25 mL), and brine, dried, and the solvent was evaporated. The crude residue was purified by column chromatography (Hex/Et_2_O, 7:3), affording **4b** (**NATxME**, 0.032 g, 18%) as a yellow dense oil. ^1^H NMR (400 MHz, CDCl_3_): δ 8.21 (d, *J* 12.0 Hz, 2 H), 8.09 (d, *J* 12.0 Hz, 2 H), 2.97–2.89 (s, 1 H), 2.79–2.71 (s, 1 H), 2.54–2.49 (s, 1 H), 2.28 (s, 3 H), 2.20 (s, 3 H), 1.98–1.89 (m, 1 H), 1.66 (s, 3 H). ^13^C NMR (125 MHz, CDCl_3_): δ 173.7, 149.3, 146.2, 139.5, 133.9, 132.1, 131.3, 121.2, 118.9, 113.1, 77.8, 52.6, 30.2, 20.5, 13.0, 11.9. HRMS m/z calcd for C_16_H_19_NO_6_ [M − H]^+^ 320.1140, found 320.1137.

### Methyl 6-((tert-butoxycarbonyl)oxy)-2,5,7,8-tetramethylchroman-2-carboxylate (**5**)

To methyl ester **1b**^27^ (1.50 g, 5.70 mmol) solution in CH_2_Cl_2_ under N_2_ flux, di-*tert*-butyl dicarbonate (1.1 g, 5.11 mmol) and 4-(dimethylamino)pyridine (0.07 g, 0.57 mmol), were added. After 2 hours of agitation at room temperature, the reaction was stopped, the solvent was evaporated, EtOAc was added (20 mL) and then washed with HCl 10% (50 mL) and saturated NaHCO_3_ (50 mL). The resulting organic layers were dried with Na_2_SO_4_, filtered and the solvent was evaporated under reduced pressure. Purification by column chromatography in SiO_2_ and Hex:EtOAc (7:3) as mobile phase rendered the desired product as a white solid (1.30 g, 65%). Analytical data are consistent with those given in the literature^33^. ^1^H NMR (400 MHz, CDCl_3_): δ 3.70 (s, 3 H), 2.69 (m, 1 H), 2.53 (m, 1 H), 2.45 (m, 1 H), 2.18 (s, 3 H), 2.11 (s, 3 H), 2.02 (s, 3 H), 1.88 (m, 1 H), 1.62 (s, 3 H), 1.57 (s, 9 H).

### 6-((tert-butoxycarbonyl)oxy)-2,5,7,8-tetramethylchroman-2-methanol (**6**)

To a suspension of LiAlH_4_ (0.07 g, 1.80 mmol) in dry THF (2 mL) cooled to 0 °C in a water-salt bath under nitrogen atmosphere, **5** (0.60 g, 1.65 mmol) dissolved in 4.0 mL of dry THF was added dropwise. The reaction mixture was stirred at 0 °C for 45 min and poured into a saturated aqueous solution of NH_4_Cl (6 mL) and extracted with EtOAc (3 × 10 mL). The combined organic layers were washed with water (1 × 10 mL), brine (1 × 10 mL), dried (Na_2_SO_4_), filtered and the solvent was evaporated under reduced pressure, to give a white solid (0.53 g, 99%). No further purification was required. Analytical data are consistent with those given in the literature^34^. ^1^H NMR (400 MHz, CDCl_3_): δ 3.70 (m, 2 H, 4 H), 2.70 (m, 2 H), 2.11 (s, 3 H), 2.10 (s, 3 H), 2.07 (s, 3 H), 2.00 (m, 1 H), 1.91 (t, 1 H), 1.76 (m, 1 H), 1.58 (s, 9 H), 1.25 (s, 3 H).

### 6-((tert-butoxycarbonyl)oxy)-2,5,7,8-tetramethylchroman-2-carbaldehyde (**7**)

To a solution of **6** (0.50 g, 1.50 mmol) in acetone, TEMPO (0.05 g, 0.32 mmol) and (diacetoxyiodo)benzene (0.65 g, 2.00 mmol) was added. After 20 h of stirring at room temperature the reaction mixture was poured into water (10 mL) and extracted with Et_2_O (4 × 10 mL). The organic layers were dried with Na_2_SO_4_, filtered and the solvent was evaporated under reduced pressure. The liquid residue was purified by column chromatography (Hex:EtOAc, 95:5) to afford a white solid (0.30 g, 60%). Analytical data are consistent with those given in the literature^49^. ^1^H NMR (400 MHz, CDCl_3_): δ 9.65 (s, 1 H), 2.67 (m, 2 H), 2.31 (m, 1 H), 2.22 (s, 3 H), 2.13(s, 3 H), 2.03 (s, 3 H), 1.87 (m, 1 H), 1.58 (s, 9 H), 1.42 (s, 3 H).

### 6-((tert-butoxycarbonyl)oxy)-2-(1-hydroxy-2-nitroethyl)-2,5,7,8-tetramethylchroman (**8**)

To a solution of imidazole (0.09 g, 1.30 mmol) in CH_3_NO_2_ (3.5 mL, 65.00 mmol), **7** (0.22 g, 0.65 mmol) was added. After 2 days of stirring at room temperature, the solvent was evaporated under reduced pressure and the crude product was poured into brine and extracted with EtOAc. The organic layers were dried with Na_2_SO_4_, filtered and the solvent was evaporated under reduced pressure. The liquid residue was purified by column chromatography (Hex:EtOAc, 8:2) to afford a white solid (0.15 g, 60%). ^1^H NMR (400 MHz, CDCl_3_): δ 4.81 (m, 1 H), 4.61 (m, 1 H), 4.44 (m, 1 H), 2.84 (d, *J* 4.0 Hz, 1 H), 2.74 (m, 2 H), 2.11 (s, 3 H), 2.09 (s, 3 H), 2.07 (s, 3 H), 1.99 (m, 2 H), 1.58 (s, 9 H), 1.26 (s, 3 H). ^13^C NMR (125 MHz, CDCl_3_): δ 77.1, 73.2, 28.2, 27.5, 19.5, 18.8, 12.8. MS (EI, 70 eV): *m/z*(%) 395(M+, 3), 334(4), 295(38), 234(37), 205(100), 189(5), 177(4).

### 2-(1-acetoxy-2-nitroethyl)-6-((tert-butoxycarbonyl)oxy)-2,5,7,8-tetramethylchroman) (**9**)

A solution of **8** (0.04 g, 0.09 mmol) in acetic anhydride (0.28 mL, 2.90 mmol,) and a catalytic amount of *p*-toluensulphonic acid, was kept in agitation for 16 h under N_2_ flow. Then, water (10 mL) was added and agitation continued for 10 more minutes to remove the excess acetic anhydride as acetic acid. The product was extracted with diethyl ether (4 mL) and the organic layer was washed with water (3 × 5 mL). The organic layer was dried with Na_2_SO_4_, filtered and the solvent was evaporated under reduced pressure to obtain a white solid (0.04 g, 93%). ^1^H NMR (400 MHz, CDCl_3_): δ 5.85 (m, 1 H), 4.88 (m, 1 H), 4.74 (m, 1 H), 2.80 (m, 1 H), 2.65 (m, 1 H), 2.14 (s, 3 H), 2.10 (s, 3 H), 2.08 (s, 3 H), 2.06 (s, 3 H), 1.90 (m, 2 H), 1.57 (s, 9 H), 1.29 (s, 3 H).

### 6-((tert-butoxycarbonyl)oxy)-2,5,7,8-tetramethyl-2-[(E)-2-nitrovinyl]chroman) (**10**)

To a solution of **9** (0.04 g, 0.09 mmol) in dry toluene, sodium carbonate was added (0.03 g, 0.09 mol); and the mixture was heated at 110 °C for 2 h. After the reaction mixture reached room temperature, it was poured into brine (10 mL) and extracted with Et_2_O (3 × 5 mL) to obtain a yellow solid (0.03 g, 94%) with no further purification. ^1^H NMR (400 MHz, CDCl_3_): δ 7.28 (d, *J* 12.0 Hz, 1 H), 7.02 (d, *J* 12.0 Hz, 1 H), 2.76 (m, 1 H), 2.57 (m, 1 H), 2.17 (s, 3 H), 2.13 (s, 3 H), 2.07 (s, 3 H), 2.05 (m, 2 H), 1.57 (s, 9 H), 1.28 (s, 3 H).^13^C NMR (125 MHz, CDCl_3_): δ 152.1, 147.9, 144.6, 141.8, 139.6, 127.9, 125.6, 122.8, 116.7, 82.9, 74.1, 31.4, 30.3, 29.7, 27.7, 25.9, 20.5, 12.7, 11.9 (2 C). MS (EI, 70 eV): m/z (%) 377 (M+, 6), 277 (100), 164 (55).

### 6-hydroxy-2,5,7,8-tetramethyl-2-[(E)-2-nitrovinyl]chroman) (**11**, **NATx0**)

To a solution of **10** (0.03 g, 0.08 mmol) in CH_2_Cl_2_, TFA (0.14 mL, 1.75 mmol) was added and the reaction was stirred for 2 h at room temperature. Then, the reaction mixture was washed with saturated NaHCO_3_ (5 mL) and brine (5 mL). The organic layer was dried with Na_2_SO_4_, filtered and the solvent was evaporated under reduced pressure. The liquid residue was purified by column chromatography (Hex:EtOAc, 8:2) to afford a yellow solid (0.02 g, 92%). ^1^H NMR (400 MHz, CDCl_3_): δ 7.28 (d, *J* 13.2 Hz, 1 H), 6.99 (d, *J* 13.2 Hz, 1 H), 4.31 (s, 1 H), 2.77 (m, 1 H), 2.56 (m, 1 H), 2.20 (s, 3 H), 2.19 (s, 3 H), 2.11 (s, 3 H), 2.09 (m, 1 H), 2.00 (m, 1 H), 1.53 (s, 3 H). ^13^CNMR (125 MHz, CDCl_3_): δ 145.5, 144.8, 144.2, 139.6, 122.3, 121.7, 118.6, 116.6, 73.7, 31.8, 26.0, 20.8, 12.2, 11.8, 11.3. HRMS m/z calcd for C_15_H_19_NO_4_ [M]^+^ 277.1314, found 277.1321.

### Buffer systems and micelles

The buffers of fixed ionic strength^41^ used for the equilibrium and kinetic measurements consisted of 20 mM Tris, 10 mM Mes and 10 mM acetic acid with 130 mM NaCl to take the ionic strength to 0.15 M and 100 µM dtpa (TMA20 buffer). Micelles of different charge were used to solubilize NATOH and NATXME, the surfactants used were sodium dodecyl sulfate (SDS), 3-[(3-cholamidopropyl) dimethylammonio]-1-propanesulfonate (CHAPS), hexa-decyltrimethylammonium bromide (CTAB) and 4-(1,1,3,3-tetramethylbutyl)phenyl-polyethylene glycol (Triton X-100). Surfactant concentrations were chosen so the concentration of micelles was actually higher than that of the nitroalkene allowing the assumption of one nitroalkene molecule per micelle. The concentrations used were 25 g/L SDS (140 µM), 10 g/L CHAPS (1.63 mM), 8 g/L Triton X-100 (101 µM), 10 g/L CTAB (450 µM), the numbers in parentheses are the micelle concentrations considering the aggregation number in each case.

### Kinetics

The reaction between the nitroalkenes and the thiols βME and GSH was followed through the change in UV absorption of the nitroalkene, at 350 nm for NATOH and NATxME or 260 nm for NATx0. The fastest reactions were monitored in a SX20 stopped-flow spectrometer (Applied Photophysics); intermediate reactions (t > 10 s) were followed in a Varian Cary50 spectrophotometer (Agilent) using a RX2000 rapid mixing stopped-flow unit (Applied Photophysics); and the slowest reactions (t > 600 s) were studied using a Varioskan Flash plate reader (Thermo). The reactions were performed in TMA20 buffer at the specified pH and 25 °C. The time courses were fitted to a single exponential function (equation 1) and the resulting rate constants (*k*_obs_) were plotted *vs* the thiol concentration to obtain the rate constants of addition (*k*_f_) and elimination (*k*_r_).

1 $${Abs}={Amp}\times \exp (-{{k}}_{{obs}}{t})+{C}$$

### p*K*_a_ determination

NATOH and NATxME in TMA20 buffer at room temperature were titrated spectrophotometrically by adding 5–10 µL of 2 M NaOH to a micellar or aqueous solution of the nitroalkene (30 µM). After each addition the pH and UV-Vis spectrum were recorded. If necessary, the initial pH was adjusted with a small volume of 2 M HCl. The absorbance readings at selected wavelengths were plotted as a function of pH and fitted to a single p*K*_a_ function (equation 2)

2 $${Abs}={A}\frac{[{{\rm{H}}}^{+}]+{{K}}_{{a}}}{[{{\rm{H}}}^{+}]}+{B}\frac{[{{\rm{H}}}^{+}]+{{K}}_{{a}}}{{{K}}_{{a}}}$$

where A and B are the absorbance of the acidic and basic forms of the nitroalkene.

### Cell culture

Murine RAW264.7 macrophages were obtained from American Type Culture Collection (ATCC® TIB-71™, Manassas, VA), maintained in complete media (DMEM containing 10% FBS, 100 U/mL penicillin and 100 μg/mL streptomycin) at 37 °C in 5% CO_2_. RAW264.7 cells were seeded the night before treatment. The next day, solutions in complete media were prepared and treated for the corresponding time points. The control group only had the media replaced at the time of treatment.

### Cell viability

Cell viability was performed by the MTT assay. Raw 264.7 macrophages were incubated with different concentrations of NATxME (3–50 μM) for 24 h. Afterwards, cell viability was assessed by measuring the mitochondrial-dependent reduction of MTT to formazan. For that purpose, MTT was added to cells to a final concentration of 0.5 mg/mL and cells were incubated at 37 °C for 1 h. After removing the media, formazan crystals were dissolved in isopropanol, and the absorbance at 570 nm was read using a microplate spectrophotometer. Results are expressed as IC_50_ (compound concentration that reduced 50% control absorbance at 570 nm). Every IC_50_ is the average of at least four determinations. (Figure S3)

### Analysis of the pro-inflammatory cytokines

RAW 264.7 cells were grown in 10% FBS/DMEM (Gibco) and then treated for 2 h with NATxME (1, 3 and 10 µM). After that, cells were stimulated with LPS (50 ng/mL) overnight. MCP-1, IL-6 and TNF-α were measured in the supernatant with a commercially available ELISA kit (BD OptEIA).

### Quantitative real-time PCR

RAW264.7 cells were grown 10% FBS/DMEM (Gibco) and then treated for 5 h with NATxME (1, 3 and 10 µM). Total RNA was extracted using TRIzol reagent (Invitrogen, Carlsbad, CA, USA). RNA was reverse-transcribed using the iScript cDNA synthesis kit (BioRad, Hercules, CA, USA) as previously described^19^. Gene expression was determined by quantitative real-time (RT)-PCR (qPCR) using TaqMan gene expression assays for heme oxygenase-1 (HMOX-1 Mm00516005_m1), GCLM (GCLM Mm00514996_m1) and NQO-1 (NQO1 Mm01253561_m1) (Applied Biosystems, Foster City, CA, USA) and normalized to GAPDH using the comparative Ct method.

### Zebrafish husbandry and inflammation assay

Zebrafish were raised and maintained according to standard protocols^50^. All experimental procedures with zebrafish were approved by the ethical committee at the Institut Pasteur de Montevideo. The neutrophil-specific zebrafish line Tg (mpx:GFP)i114, referred to as Tg(mpx:GFP), was used for all inflammation assays, and neutrophil tracking assays at 3 dpf as previously described^36^. In all inflammation assays, larvae were preincubated for 2 h with compounds at the doses indicated in each figure. Tail fins were transected with a sterile scalpel at the region indicated in Fig. 4A and larvae were incubated in the presence of compounds for 4 h, and fixed in 4% paraformaldehyde overnight. To assess neutrophil number, whole mount immunohistochemistry was performed as described^51^ using rabbit polyclonal anti-GFP (Invitrogen A-11122). Images were captured in an inverted fluorescence microscope (Olympus) at 10x magnification, and neutrophils in the region posterior to the circulatory loop were counted (Fig. 4B).

## Electronic supplementary material

Supplementary information
