# Supplementary material for: Electrophilic nitroalkene-tocopherol derivatives: synthesis, physicochemical characterization and evaluation of anti-inflammatory signaling responses
Source: Sci Rep. 2018 Aug 24;8:12784. doi: 10.1038/s41598-018-31218-7 (PMC6109136; doi:10.1038/s41598-018-31218-7)
Supplement: Supplementary file 1 — Supplementary information [file 41598_2018_31218_MOESM1_ESM.pdf]

Supplementary Information

Electrophilic nitroalkene-tocopherol derivatives: synthesis, physicochemical characterization and evaluation of anti-inflammatory signaling responses

Jorge Rodriguez-Duarte[1,2,7], Rosina Dapueto[1,2,7], Germán Galliussi[1,7], Lucía Turell[3], Andrés Kamaid[4,7], Nicholas K.H. Khoo[5], Francisco J. Schopfer[5], Bruce A. Freeman[5], Carlos Escande[6,7], Carlos Batthyány[1,4,7,#], Gerardo Ferrer-Sueta[7#], Gloria V. López[1,2,7,#],

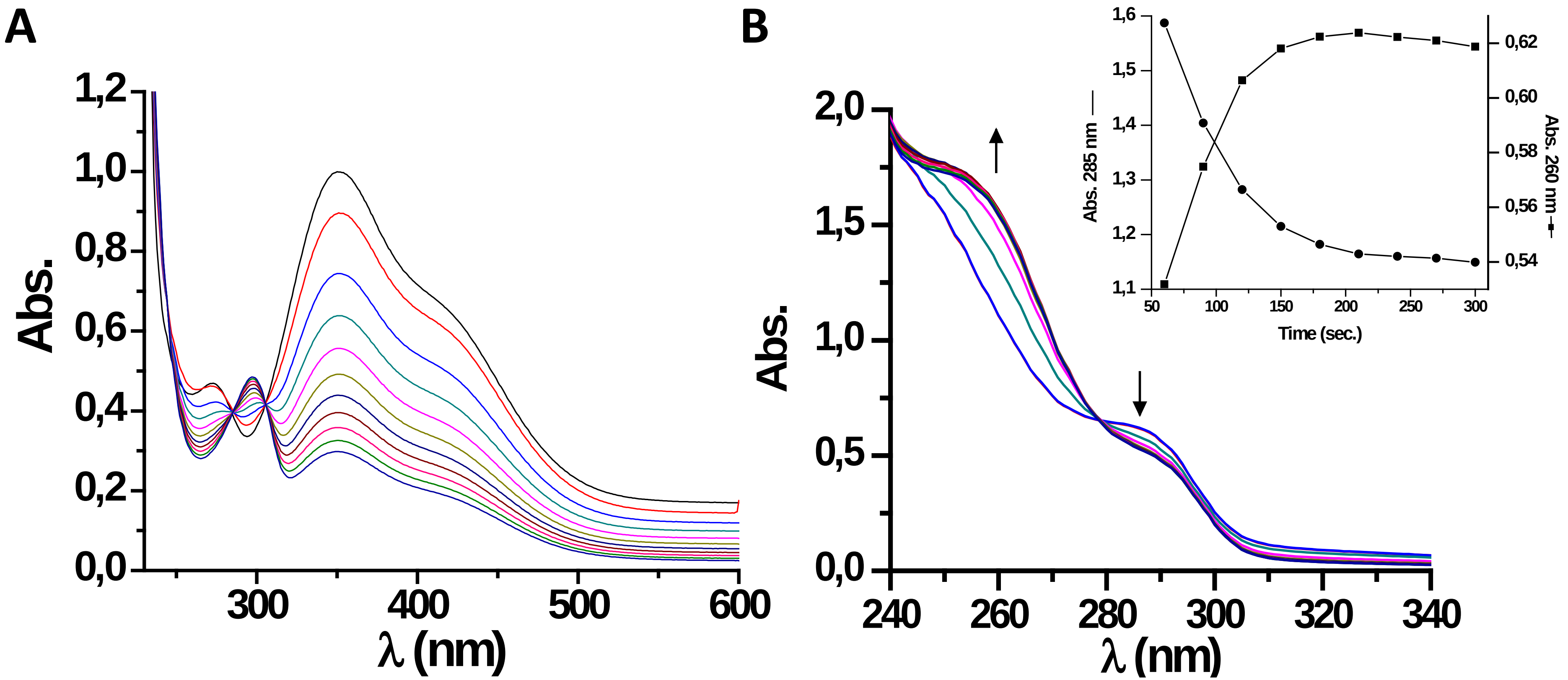

Figure S1. **A.** Reaction of NATxME with BME. Spectral change upon reaction of 100 μM NATxME with BME (1 mM) in phosphate buffer, 20 mM, pH 7.4. Spectra were obtained every minute (240 – 600 nm range). **B.** Reaction of NATx0 with BME. NATx0 (130 μM) in phosphate buffer (20mM) pH 7.4 was incubated with BME (1mM). Spectra of the reaction were obtained every 60 s (240–340 nm range).

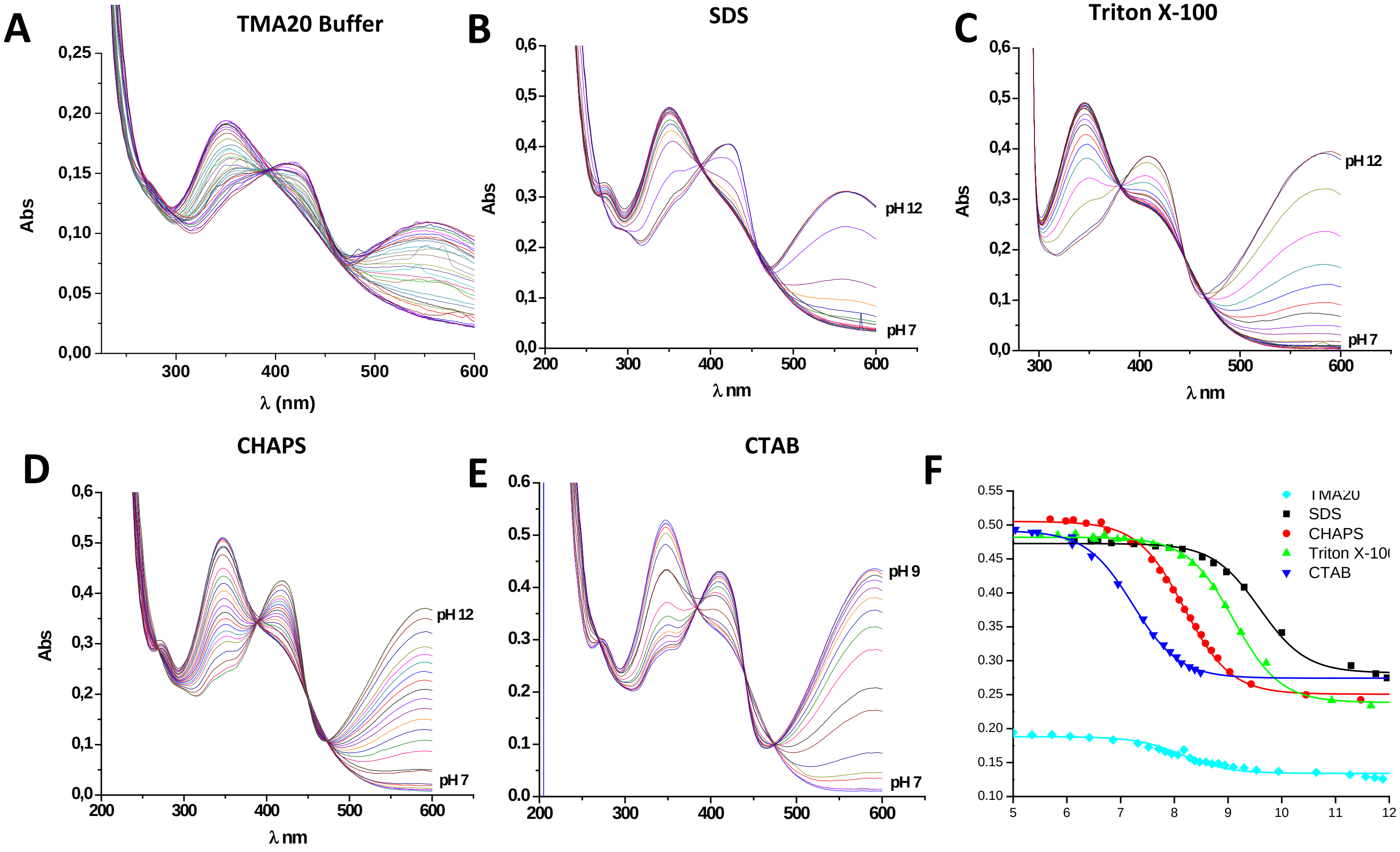

Figure S2. NATxME titration in aqueous solution with or without different detergents. NATxME (30 µM) in TMA20 buffer with or without different detergents was titrated by adding 5-10 µl of 2M NaOH and HCl. **(A):** TMA20 buffer; **(B)** SDS 0.25%; **(C):** Triton X-100 1%; **(D):** CHAPS 1%; **(E):** CTAB 1%. **(F):** Titration curves of NATxME at 350 nm in TMA20 buffer and different micelle suspensions, from left to right, CTAB, TMA20 buffer, CHAPS, Triton X-100 and SDS. The p*K*a was determined from the sigmoidal fitting of the plot Abs 350 vs pH. CTAB: 7.3; TMA buffer: 7.96; CHAPS: 8.18; Triton X-100:9.1; SDS: 9.58

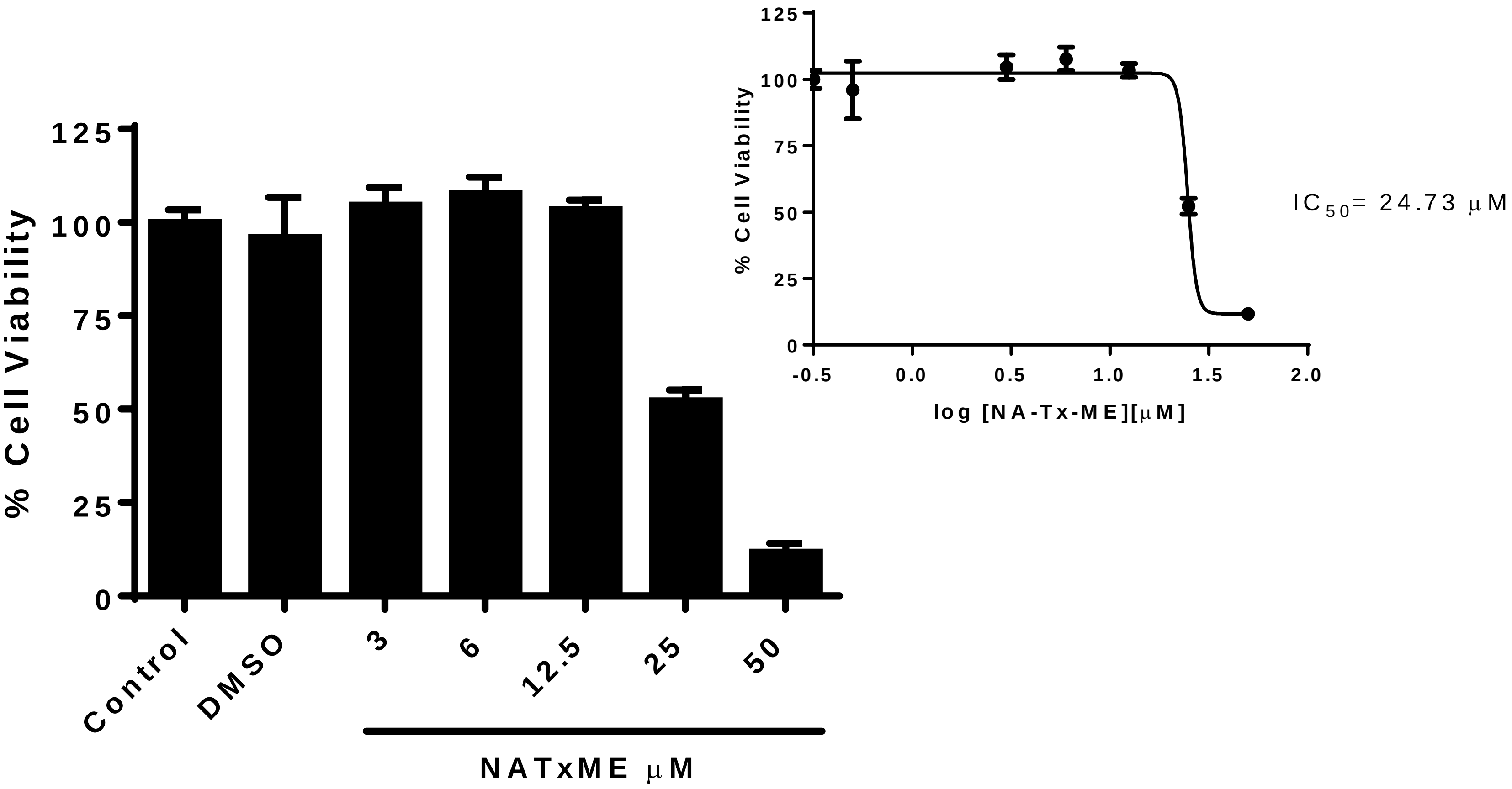

Figure S3. NATxME cell viability. Raw 264.7 macrophages were incubated with different concentrations of NATxME (3-50mM) for 24hs. Cell viability was measured by using the MTT assay. Results are expressed as IC50 (compound concentration that reduced 50% control absorbance at 570 nm). Every IC50 is the average of at least four determinations.

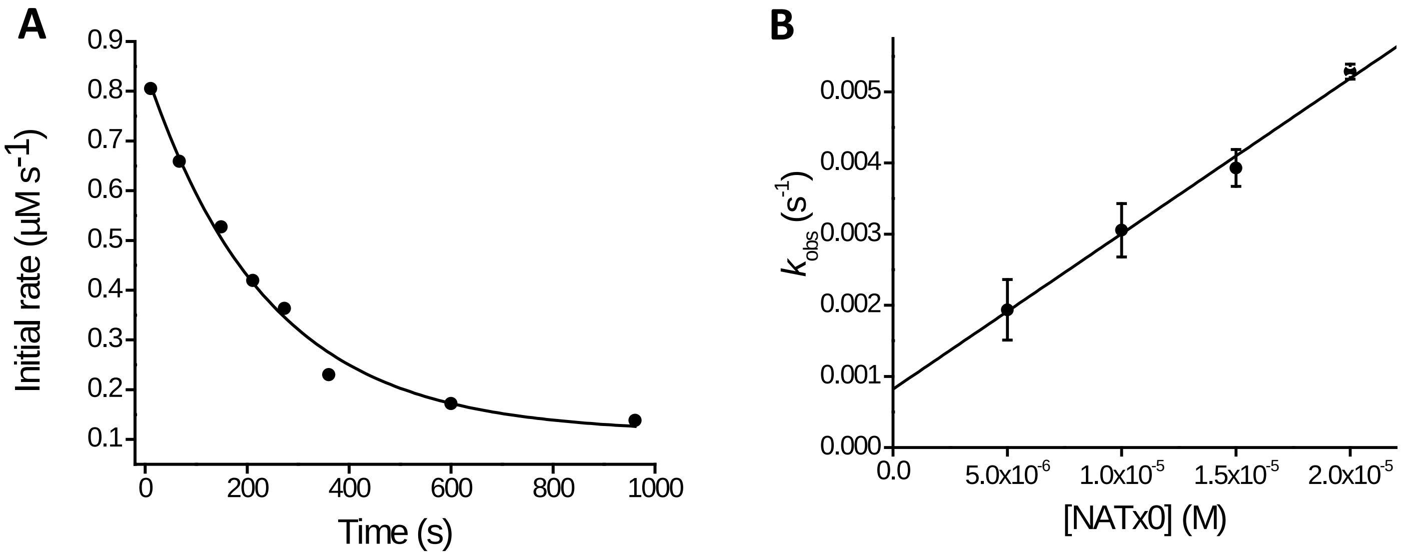

Figure S4. Reaction of NATx0 with GAPDH. (A) Reduced GAPDH (1 μM) was incubated with 15 μM NATx0 (25°C, Tris buffer, 0.1 M, pH 7.4, 0.1 mM DTPA). At increasing times, aliquots (0.1 μM) were mixed with 0.5 mM GAP, 1 mM NAD^+ and 15 mM sodium arsenite, and the initial rate of NADH formation at 340 nm and 25 °C was measured. The solid line represents the best fit to a single exponential equation. **(B)** k_{obs} values at increasing concentrations of NATx0 (5 - 20 μM) were determined from kinetic traces as in (A).
